# Who and how to screen for endogenous hypercortisolism in adrenal and pituitary incidentaloma

**DOI:** 10.1007/s40618-024-02456-6

**Published:** 2024-10-12

**Authors:** Kimberly Coscia, Martina Verrienti, Guido Di Dalmazi, Maria Chiara Zatelli

**Affiliations:** 1 Division of Endocrinology and Diabetes Prevention and Care, Department of Medical and Surgical Sciences (DIMEC), IRCCS Azienda Ospedaliero-Universitaria di Bologna, Alma Mater Studiorum University of Bologna, Bologna, Italy; 2 Department of Medical and Surgical Sciences (DIMEC), Alma Mater Studiorum University of Bologna, Bologna, Italy; 3 Section of Endocrinology, Geriatrics and Internal Medicine, Department of Medical Sciences, University of Ferrara, Ferrara, Italy

**Keywords:** Adrenal incidentalomas, Pituitary incidentalomas, Hypercortisolism, Cushing’s syndrome, Cushing’s disease

## Abstract

**Purpose:**

Adrenal incidentalomas (AIs) and pituitary incidentalomas (PIs) have become frequent findings in the last two decades due to the widespread use of cross-sectional imaging in clinical practice. This review investigates the prevalence of endogenous hypercortisolism in patients with AIs and PIs. We aim to underscore the importance of early detection and management of endogenous hypercortisolism in this subset of patients to mitigate associated cardiometabolic complications and reduce mortality.

**Methods:**

We performed a PubMed literature search to provide updates regarding the prevalence of endogenous hypercortisolism in patients with AIs and PIs, the demographic and clinical characteristics of the studied populations, and the diagnostic test accuracy for early identification of endogenous hypercortisolism.

**Results:**

Hypercortisolism, especially mild autonomous cortisol secretion (MACS), was identified in a notable proportion of patients with AIs. MACS was associated with increased cardiometabolic risks, contributing to an elevated overall mortality rate in this cohort. Furthermore, PIs were found to be linked with Cushing’s disease in a subset of patients, emphasizing the need for thorough evaluation and monitoring.

**Conclusion:**

Early diagnosis and appropriate management of endogenous hypercortisolism are essential in preventing complications and improving patient outcomes. As the presence of undetected hypercortisolism is associated with clinical complications over time, the accurate identification of high-risk populations to screen remains crucial.

## Introduction

Current clinical practice employs imaging studies more and more frequently for diagnostic purposes of diseases involving the cranial and abdominal districts. As a consequence, the incidence of adrenal incidentalomas (AIs) and pituitary incidentalomas (PIs) significantly increased. The aim of this review is to investigate the prevalence of endogenous hypercortisolism in patients with AIs and PIs, to foster early diagnosis and prevent important complications.

## Methods

We performed an extensive literature review using Pubmed, from January 1990 to October 2023. The following keywords were included: “hypercortisolism, adrenal incidentaloma, pituitary incidentalomas, hypercortisolism, Cushing’s syndrome, and Cushing’s disease”. Search terms were linked to the Medical Subject Headings (MeSH) when possible. Keywords and free words were used simultaneously. Additional articles were identified with manual searches and included thorough review of other meta-analyses, review articles, and relevant references.

### Adrenal and pituitary incidentalomas: definition and epidemiology

Adrenal incidentalomas (AIs) are defined as clinically silent adrenal masses (diameter ≥ 1 cm) detected incidentally during radiological studies performed for issues unrelated to adrenal diseases [1]. Adrenal masses discovered during screening imaging performed for extra-adrenal tumor workup or hereditary syndromes associated with adrenal tumors are not included in the definition of AIs [1, 2]. Due to the rising number of cross-sectional imaging studies performed in clinical practice, AIs have become a rather common finding in the last two decades. As underlined in a large population-based cohort study, incidence rates of adrenal tumors have increased 10 times from 1995 to 2017 [3]. However, information about epidemiology of AIs varies depending on the type of data source (e.g., autopsy, surgery, or radiology series) [4]. The overall prevalence of AIs is estimated to be about 3-4.2% among all imaging studies, whereas it varies from 1.1 to 8.7% in autopsy series (Table 1) [4, 5]. The prevalence of AIs increases up to 10% in the older population (> 70 years old), with an incidence peak between the fifth and the seventh decade, equally distributed between males and females [4, 6]. On the other hand, AIs represent a rare finding during childhood and adolescence, accounting for 0.3–0.4% of all tumors in children [7]. Prevalence of AIs is also higher in the Caucasian population and increases considering patients affected by obesity, type 2 diabetes, and hypertension [8]. The differential diagnosis of adrenal masses includes benign and malignant tumors. AIs are frequently unilateral, while bilaterality is reported in 15% of patients [6, 9, 10]. However, the prevalence of bilateral AIs might be higher when autonomous cortisol secretion is present [11, 12]. Although most AIs are benign, asymptomatic, and nonfunctioning (70–80%), ruling out malignancy is mandatory, as adrenocortical carcinomas might be found in 2–5% of the cases [13]. At baseline evaluation, adrenal function should also be assessed since the rate of hormonally active AIs ranges between 11 and 25%, thus determining relevant clinical features [3]. Cortisol excess represents the most frequent hormonal impairment among patients with secreting AIs. About 20% of the cases of Cushing’s syndrome (CS) might be due to adrenal tumors, even though the overall incidence of the disease remains low among the general population (2–5%) [14]. The prevalence of mild autonomous cortisol secretion (MACS) in AIs varies depending on the thresholds used in the biochemical assessment. In a recent retrospective multicenter study of patients with AIs, the 1-mg post-dexamethasone cortisol threshold of 50 nmol/L was associated with a prevalence of MACS of 33.5%. By increasing this threshold to 83 nmol/L and 138 nmol/L respectively, the prevalence decreases to 13.7% and 5.6% [15]. Frequency of MACS is consequently higher when using the lowest threshold of 1 mg overnight dexamethasone suppression test (ODST), as reported in selected series [11, 16]. In addition, MACS may develop in patients with initial nonfunctioning tumors, as a result of progression of hormonal secretion over time [2, 17, 18]. A recent meta-analysis showed that the overall prevalence of secreting AIs was 27.5%, with cortisol-secreting tumors representing the majority of functioning AIs (approximately 12–20%), followed by aldosterone-secreting tumors (3–6%), and pheochromocytomas (3–5%) [13, 19, 20]. CS was found in about 2–4% of AIs [20]. Moreover, the prevalence of cortisol-secreting AIs was higher in Europe and America [20]. Table 1 provides a summary of the general occurrence of functioning AIs. Most AIs associated with endogenous hypercortisolism are represented by benign adrenocortical adenomas, but bilateral adrenal hyperplasia and adrenocortical carcinomas might represent other possible causes.

**Table:** Table 1

**Prevalence of adrenal incidentalomas**
Imaging studies 3–10%
Autopsy studies 1.05–8.7%
**Prevalence of hormonally active adrenal incidentalomas**
Mild Autonomous Cortisol Secretion 5.6–33.5%
Primary aldosteronism 3–6%
Pheocromocytoma 3–5%
Cushing’s syndrome 2–4%
**Characteristics of high-risk populations**
Females
Young age (< 65 years)
Multiple and progressive signs and symptoms of hypercortisolism
Adrenal incidentaloma compatible with benign adrenocortical adenoma

*References:* [1, 3–5, 13, 14, 17, 19, 20].

Similar to AIs, pituitary incidentalomas (PIs) are lesions incidentally discovered in the pituitary gland during brain imaging studies performed for unrelated reasons (e.g., headache, head trauma, neurological or ENT complaints). Usually, PIs are small (< 1 cm in size) and not associated with overt symptoms related to hormone overproduction or mass-effect. They are relatively common findings in brain imaging studies, with a higher prevalence in older adults and a slight predominance in women. The prevalence is 15–21/100.000 in the general population, representing 16-36% of pituitary adenomas (PAs) [21–23]. The prevalence may vary depending on the background population and the sensitivity of the imaging techniques. In the US, PIs incidence rate increased from 0.73 ± 0.05 to 2.00 ± 0.09 per 100,000 between 2004 and 2018, mostly in females, showing the highest incidence in patients ≥ 80 years [23]. However, this study does not report patients’ clinical characteristics, therefore the percentage of patients with Cushing’s disease (CD) is unknown. A recent review reported that the prevalence of functioning adenomas among PIs is 8.8–25%, with a high prevalence of macroadenomas (49–84%) [24]. 

PIs usually are benign lesions, even though they may require careful evaluation and management, to accurately determine their growth potential, and impact on nearby structures by neuroimaging (contrast-enhanced MRI). Frequent causes differ according to age. In adults, PIs are mainly represented by pituitary adenomas, followed by Rathke cleft cysts. Other entities (craniopharyngioma, meningioma, metastases, hypophysitis, or infiltrative diseases) are rare [25, 26]. In children, PIs are more frequently due to the presence of a Rathke cleft cyst or other cystic lesions. In a paediatric cohort (mean age: 11 ± 6 years) PI incidence was overall 2,2%; incidental lesions were almost twice as frequent in the older age groups (10–18 years) as compared to younger patients and in females. These findings might be explained by the indications for imaging in this paediatric cohort (growth impairment and headache). Nevertheless, pituitary adenomas in these settings are rare [27]. In addition, PI identified during childhood tend to remain stable in size [28]. 

PIs should be investigated for prolactin (PRL) and growth hormone (GH) hypersecretion by evaluating basal PRL and/or IGF-1 levels in all patients and for CD in clinically suspected patients. Indeed, significant comorbidity is associated with undiagnosed hyperprolactinemia, acromegaly and CD. An Italian study screened 68 PI patients for CD by performing a 1 mg dexamethasone suppression test, urinary free cortisol and midnight serum or salivary cortisol assessment, showing a 7% prevalence of ACTH dependent hypercortisolism [29]. In different settings, very rarely Cushing’s disease (CD) was diagnosed among incidentally discovered pituitary adenomas [30]. However, most of the available studies reporting on PI associated with CD are observational retrospective studies. Therefore, further studies are required to fill that gap.

Interestingly, rare cases of CD associated with non-functioning AIs were described as well [31, 32].

### Adrenal and pituitary incidentalomas: clinical presentation of endogenous hypercortisolism and at-risk population

CS encompasses several clinical manifestations resulting from excessive and prolonged exposure of body tissues to glucocorticoids. Typical signs and symptoms of CS, such as abdominal obesity, facial plethora, muscle weakness, proximal myopathy, skin fragility, and abdominal purple striae, might be clinically evident in overt hypercortisolism [33]. As pointed out by a recent study based on data from the German Cushing registry, the presence of multiple (> 3) and progressive signs and symptoms suggestive of hypercortisolism, especially if unusual for patient’s age, increases the likelihood of endogenous hypercortisolism detection [33]. Based on the chief complaints, the same study revealed a significantly higher occurrence of AIs in patients diagnosed with CS. Additionally, the proportion of confirmed CS reaches a percentage of 40% in the group of patients with AIs in selected referral centers [33]. 

MACS is defined as ACTH-independent overproduction of glucocorticoids in AI, in patients without a clear cushingoid phenotype [34]. Despite the absence of catabolic signs of CS, patients with MACS frequently present metabolic and cardiovascular complications [19, 35]. The prevalence of diabetes in patients with MACS is about 30% and increases up to 70% in CS [36]. Hypertension is found in about 80–85% of patients with CS and often occurs as initial presentation of the disease [14]. Furthermore, several retrospective studies have demonstrated that the prevalence of cardiovascular and thromboembolic events is higher in patients with cortisol-secreting adrenal masses than those with nonfunctioning tumors, thus resulting in increased overall mortality in the group of cortisol-secreting AIs, which seems to be higher in women younger than 65 years [12, 19, 37]. The prevalence of vertebral fractures in patients with CS is up to 76% and most of these patients show lower trabecular bone scores associated with only mildly reduced bone mineral density [38]. A recent large cross-sectional study illustrated that MACS in AIs is associated with an increased risk of fragility fractures, especially in post-menopausal women over 65 years old [39]. Notably, patients with benign adrenal tumors diagnosed with MACS or CS experience more severe hypertension, a higher incidence of type 2 diabetes, and a greater need for insulin therapy to control their blood sugar levels compared to those with non-functioning adrenal tumors, as pointed out in a recent cross-sectional study [40]. Table 1 highlights the characteristics of the population at high-risk of CS. Considering the most pertinent studies, the prevalence of MACS and CS in AIs, along with their clinical presentation, was reported in Table 2.

**Table 2:** Prevalence of MACS and CS in AIs, demographics, clinical presentation, and screening test thresholds in relevant studies

Article	No of patients	CS prevalence		MACS prevalence	Gender No F/M	Median or mean Age	Clinical presentation of MACS and/or CS	Screening test	Threshold
**Barzon et al. (2002)**[18]	284	*N* = 4 (1.4%)	*N* = 32 (11.2%)		**Total** 170/114 **CS** 4/-	56	CS: skin atrophy, easy bruising, moon face, ecchymoses, depression, osteoporosis, overweight, hypertension, muscle weakness.	MACS: 1 mg-DST	Cortisol DST ≥ 138 nmol/L
**Masserini et al. (2009)**[62]	103	-	*N* = 22 (21.3%)		**Total** 69/74	62	Hypertension (*N* = 11); dyslipidaemia (*N* = 5); diabetes mellitus (*N* = 6).	MACS: LNSC with IFMA MACS: 1 mg-DST MACS: Morning ACTH MACS: UFC	5.1 nmol/L Cortisol DST > 83 nmol/L ACTH < 2.2 pmol/l UFC > 193 nmol/24 h.
**Nunes et al. (2009)**[64]	48	*N* = 3 (6.2%)	*N* = 23 (48%)		**Total** 31/17	-	-	MACS: LNSC with RIA MACS: 1 mg-DST	4.8 nmol/L Cortisol DST > 60 nmol/L
**Reimondo et al. (2011)**[55]	68	-	*N* = 22 (32%)		**Total** 45/23	58	Hypertension (*N* = 24), impaired fasting glucose/diabetes mellitus (*N* = 8), obesity (*N* = 6).	MACS: 1 mg-DST	Cortisol DST > 138 nmol/L Cortisol DST 50–138 nmol/L
**Ceccato et al. (2017)**[60]	164 Ais + 46 CS	-	*N* = 30 (18.2%)		**Total** 92/72	63	-	CS: 1 mg-DST CS: NSC CS: UFC	Cortisol DST > 138 nmol/L Cortisol DST > 50 nmol/L 14.46 nmol/L 170 nmol/24 h
**Ceccato et al. (2018)**[16]	106	-	*N* = 46 (43%)		**Total** 61/45	66	-	MACS: LNSC with LC-MS/MS	1.1 nmol/L
**Ponzetto et al. (2020)**[50]	50	*N* = 14 (11 CD, 3 CS)	*N* = 12 (24%)		**Total** 39/11	18–61	-	CS: LNSC with LC-MS/MS	3.17 nmol/L
**Kjellbom et al. (2021)**[11]	1048	-	*N* = 82 (7.8%) *N* = 391 (37.3%)		**MACS** 291/182	69	Hypertension (*N* = 57); diabetes mellitus (*N* = 14); cardiovascular disease (*N* = 22); heart failure (*N* = 7).	MACS: 1 mg-DST	Cortisol DST ≥ 138 nmol/L Cortisol DST 50–137 nmol/L
**Deutschbein et al. (2022)**[12]	3656	-	*N* = 247 (6.8%) *N* = 320 (36.1%)		**MACS** 1029/538	61	Hypertension (*N* = 1123); dyslipidaemia (*N* = 670); diabetes mellitus (*N* = 350); cardiovascular event (*N* = 169).	MACS: 1 mg-DST	Cortisol DST > 138 nmol/L Cortisol DST 50–138 nmol/L
**Prete et al. (2022)**[40]	1305	*N* = 65 (4.9%)	*N* = 451 (34.5%) *N* = 240 (18.3%)		**MACS** 406/285 **CS** 56/9	60	Hypertension (MACS *N* = 446; CS *N* = 47); type 2 diabetes (MACS *N* = 192; CS *N* = 20); dyslipidemia (MACS *N* = 191; CS *N* = 10).	MACS:1 mg-DST	Cortisol DST > 138 nmol/L Cortisol DST 50–138 nmol/L
**Remde et al. (2022)**[35]	260	-	*N* = 41 (15.8%) *N* = 96 (36.9%)		**Total** 147/113	60	Hypertension, diabetes mellitus, dyslipidemia, and obesity.	MACS: 1 mg-DST	Cortisol DST > 138 nmol/L Cortisol DST 50–138 nmol/L

*Abbreviations*: MACS, mild autonomous cortisol secretion; CS, Cushing’s syndrome; AIs, adrenal incidentalomas; 1 mg-DST, 1 mg - dexamethasone suppression test; Cortisol DST, cortisol post dexamethasone suppression test; LNSC, late-night salivary cortisol; LC-MS/MS, liquid chromatography-tandem mass spectrometry; UFC, 24 h urinary free cortisol. IFMA, immuno-fluorimetric assay; RIA, radioimmunoassay; ECLIA, electrochemiluminescence immunoassay

Therefore, when multiple and progressive signs (especially catabolic signs) and symptoms related to hypercortisolism are observed in patients with AIs and PIs, it is essential to rule out CS. Personal and pathological history, physical examination, and evaluation of photographs taken years before are useful to detect signs and symptoms that may suggest the presence of hypercortisolism. Patients younger than 40 with unjustified weight gain, facial fullness, plethora, hirsutism, proximal myopathy, dorsocervical fat pad, hypertension, diabetes, and low bone mineral density may be at higher risk and therefore should be screened for endogenous hypercortisolism according to the proposed “Cushing score” [41]. A previously proposed risk scoring system included muscular atrophy, osteoporosis, dorsocervical fat pad together with late-night salivary cortisol evaluation to assess the probability of hypercortisolism in an outpatient setting [42]. However, these scores have not been validated in independent studies and there is no evidence that they could be usefully applied in the settings of AIs and PIs. Nevertheless, a recent study showed that patients presenting with myopathy, metabolic syndrome, osteoporosis, adenoma, and multiple CS-specific symptoms (muscle weakness, easy bruising, persistent high blood pressure, uncontrolled or difficult-to-manage diabetes, unexplained osteoporosis or fractures, severe depression or mood disorders that are unresponsive to treatment, women with menstrual irregularities or hirsutism) have a high likelihood of CD and should therefore be screened for endogenous hypercortisolism. On the contrary, obesity alone does not seem to be very indicative of CD [33]. In addition, ACTH secretion and consequent cortisol levels have been reported to correlate with pituitary adenoma diameter, therefore, being PIs usually small, e milder phenotype can be expected in PI vs. AI [43]. It is important to point out that PIs may be associated with other conditions, such as multiple endocrine neoplasia type 1 (MEN1) or familial isolated pituitary adenoma syndromes, therefore investigation of family history is also relevant. On the other hand, the correct identification of patients with MACS based on clinical grounds could be challenging in patients with AIs, because many clinical manifestations of hypercortisolism, such as hypertension, diabetes, and osteoporosis, are commonly detected in the general population [33, 44]. For these reasons, the latest European Society of Endocrinology clinical practice guidelines stated that all patients with a new diagnosis of AI should undergo biochemical evaluation to rule out MACS [1]. Such a biochemical screening could be avoided in elderly patients with poor clinical conditions and limited life expectancy [1]. 

### Biochemical screening of endogenous hypercortisolism in adrenal incidentalomas and pituitary incidentalomas

The use of exogenous glucocorticoids should be excluded before any further evaluation. The biochemical screening for endogenous hypercortisolism applies to unilateral and bilateral AIs, as well as PIs. CD consensus guidelines for testing should be followed to screen patients with PIs and a high pre-test probability of CD [45]. According to the clinical practice guidelines, the initial biochemical screening of CS should assess the proper function of the hypothalamic-pituitary-adrenal (HPA) axis with one of the following tests: 24-h urinary free cortisol (UFC) in at least two measurements; 1-mg ODST; late-night salivary cortisol (LNSC) in at least two measurements [46]. Clinicians should select the most appropriate test considering the patient’s specific characteristics and the main pitfalls of each screening test.

The 1-mg ODST is influenced by several conditions, resulting in both false-negative and positive results. Firstly, clinicians should always ensure the adherence of patients and the correct assumption of dexamethasone tablets. False-negative results might be seen after exogenous glucocorticoid exposure, as it represents the main interference on the HPA axis. For this reason, clinicians should carefully exclude corticosteroid use before screening patients with AIs. Other concurrent medications (e.g., fluoxetine, cimetidine, diltiazem) might lead to false-negative results, because of the inhibition of the hepatic CYP3A4 complex, responsible for dexamethasone metabolism. Additionally, patients with concurrent nephrotic syndrome may exhibit decreased concentrations of cortisol binding globulin (CBG) and albumin, which can lead to falsely low values [45]. On the other hand, CYP3A4 inducers, such as phenobarbital, carbamazepine, or St. John’s wort, might cause false-positive results. Similarly, dexamethasone malabsorption due to gastrointestinal diseases, elevated concentrations of CBG induced by oral estrogens, pregnancy, or chronic active hepatitis might lead to the same result [45]. Measuring plasma levels of dexamethasone may clarify the presence of potential abnormalities in dexamethasone clearance [47]. However, this assay is not widely available yet. UFC can mainly be affected by incorrect collection of urine samples, increased water loading, salt restriction, and renal impairment. Moreover, the lack of gender-specific reference ranges and the limited access to the more accurate liquid chromatography-tandem mass spectrometry (LC–MS/MS) method may hinder the correct interpretation of results [48–50]. LNSC presents high diagnostic accuracy to exclude hypercortisolism in the presence of normal cortisol levels, even when measured with chemiluminescent immunoassays [51]. However, in certain individuals, such as night workers, LNSC should not be performed due to the disruption of the circadian rhythm of cortisol secretion. Additionally, LNSC may not be useful in smokers and patients with insufficient saliva for analysis and/or oral diseases. By contrast, LNSC remains a valuable screening tool for CS in women with elevated corticosteroid-binding globulin due to estrogen therapy or pregnancy [45]. 

Table 2 displays the thresholds for first-line screening tests for endogenous hypercortisolism in AIs reported in the most relevant studies. When CS caused by an adrenal tumor is suspected, the recommended initial test is 1-mg ODST [45]. If the initial testing shows normal cortisol levels, hypercortisolism can be excluded. However, if there is a high likelihood of CS based on the patient’s clinical picture or if symptoms worsen within six months, reevaluation may be recommended [46]. To diagnose CS after an abnormal initial result, at least one of the remaining screening tests must be positive, [14] keeping in mind that more than one test should be performed to increase diagnostic specificity [52]. Therefore, a combination of multiple tests should be performed to confirm the diagnosis of CS. ACTH levels need to be evaluated to determine the cause of CS, as adrenal tumors lead to ACTH-independent CS with suppressed/low ACTH levels [46]. Dynamic tests, such as dexamethasone-corticotropin releasing hormone (Dex-CRH) test, Desmopressin test, and CRH test, are useful to distinguish between neoplastic and non-neoplastic hypercortisolism [53], and are typically performed as second-line evaluations in specialized centers. Nevertheless, the current shortage of CRH represent an important limitation in the use of those tests [54]. 

If cushingoid stigmata are absent in patients with AI, MACS should be excluded by using 1-mg ODST as a first-line screening test, due to its ease of use and availability in clinical practice [1]. Post-dexamethasone cortisol values below 50 nmol/L (1.8 µg/dL) can effectively rule out MACS, despite the absence of a clear consensus regarding the specific thresholds to be utilized [8, 55]. Several studies have reported that post-dexamethasone serum cortisol levels above 50 nmol/L provide reliable biochemical evidence of cortisol autonomy, demonstrating a sensitivity of nearly 100% and a high negative predictive value [48]. However, the recommended cutoff points are characterized by low diagnostic specificity (about 80%), thus leading to frequent false-positive results [56]. For this reason, all patients at the first screening should be retested to confirm MACS. A further stratification of patients based on post-dexamethasone cortisol values is no more recommended, as MACS should be considered a biological continuum in terms of both hormonal and cardiometabolic complications. Furthermore, the diagnostic accuracy of the 1-mg ODST for the prediction of cardiometabolic comorbidities in patients with AIs could be inadequate [15]. Post-dexamethasone cortisol values above 50 nmol/L identify patients with MACS as a high-risk population with increased overall mortality, but treatment should be individualized based on the presence of cardiometabolic comorbidities. In this scenario, longitudinal monitoring through serial testing should be performed to observe any trends in cortisol levels or clinical changes, as it can be useful for treatment decision-making (e.g., adrenalectomy or conservative approach). Consideration should also be given to the radiological characteristics of adrenal tumors since larger adrenal incidentalomas (≥ 28 mm) had over a tenfold increased risk of developing adrenal hypercortisolism during the follow-up period compared to smaller lesions. Bilateral incidentalomas were also associated with a higher prevalence of MACS [57]. In cases of MACS, the adrenal lipid content may be influenced by the functional state, which can, in turn, affect the pre-contrast CT attenuation values. A recent study found that most benign cortisol-secreting adenomas (approximately 79%) had a pre-contrast attenuation value greater than 10 HU, with 64.7% showing a value greater than 20 HU [58]. These findings align with the majority of cortisol-secreting adenomas being lipid-poor. The lack of specificity of 1-mg ODST indicates that further biochemical assessments might be required for the screening of MACS, even though none of the tests recommended for diagnosis of CS are sufficiently reliable in patients with MACS. Basal morning ACTH values are useful for assessing ACTH independency, as patients often show low-normal or suppressed ACTH values. However, the performance of currently available ACTH assays might not be sufficiently reliable, especially in the interpretation of low ACTH values [59]. Additionally, UFC and LNSC can provide complementary biochemical information in the presence of clinical features highly suspicious of hypercortisolism, but it should be noted that they lack both sensitivity and specificity when used alone [4]. UFC has a limited ability to identify MACS, as free cortisol is detectable in urine only when its concentration exceeds the plasma binding capacity [60]. Thus, impaired UFC values usually represent a late finding of emerging CS. LNSC demonstrates limited sensitivity in the diagnosis of MACS since the circadian rhythm of cortisol is often conserved in those patients [16, 61, 62]. On the other hand, patients with CS loose the normal circadian nadir of cortisol secretion, enhancing the utility of LNSC as a sensitive and specific screening tool for overt CS [63, 64]. Nevertheless, recent data suggest using mass spectrometry for further patient stratification in AIs based on steroid profiling [65]. Furthermore, a recent prospective study proposed DHEAS measurements as a simple screening biomarker for MACS. Among 287 patients with AIs (45 with MACS and 242 with non-functioning AI), low DHEAS levels (< 60 µg/dl) showed a sensitivity of 75.6% and specificity of 81.4% for MACS detection [66]. However, recommendations for the use of serum DHEAS for MACS screening are debated due to limited evidence, thus further multicenter studies are required to validate these findings.

Ultimately, the decision to screen for hypercortisolism should be based on a comprehensive evaluation by a healthcare professional, considering individual risk factors and clinical presentation.

## Conclusions

In conclusion, endogenous hypercortisolism should be excluded in all patients with a newly diagnosed AI and PI, especially when the clinical picture typical of CS is observed. In the absence of cushingoid stigmata, the biochemical screening gains great clinical relevance because of the high prevalence of MACS among AIs and the consequent increase in overall mortality in this population. Due to its high sensitivity, availability, and ease of use, the 1-mg ODST is recommended as initial screening test. Throughout the screening process, the expertise of clinicians remains crucial in understanding the diagnostic performance of different biochemical tests and evaluating the diverse range of clinical manifestations associated with endogenous hypercortisolism.

Patients with PIs and a high pre-test probability of CD should be screened as well, especially when their clinical history and symptoms indicate a potential risk factor.

## Declarations﻿

### Statements and declarations

The Authors have nothing to disclose.
