# Correction: Who and how to screen for endogenous hypercortisolism in adrenal and pituitary incidentaloma

**DOI:** 10.1007/s40618-024-02489-x

**Published:** 2024-11-09

**Authors:** Kimberly Coscia, Martina Verrienti, Guido Di Dalmazi, Maria Chiara Zatelli

**Affiliations:** 1 Division of Endocrinology and Diabetes Prevention and Care, Department of Medical and Surgical Sciences (DIMEC), IRCCS Azienda Ospedaliero-Universitaria di Bologna, Alma Mater Studiorum University of Bologna, Bologna, Italy; 2 Department of Medical and Surgical Sciences (DIMEC), Alma Mater Studiorum University of Bologna, Bologna, Italy; 3 Section of Endocrinology, Geriatrics and Internal Medicine, Department of Medical Sciences, University of Ferrara, Ferrara, Italy

**Correction:** **Journal of Endocrinological Investigation** 10.1007/s40618-024-02456-6

In the original version of this article, some part of the Funding information was missing. The funding information was previously read “Open access funding provided by Alma Mater Studiorum - Università di Bologna within the CRUI-CARE Agreement” Should have been “Open access funding provided by Alma Mater Studiorum - Università di Bologna within the CRUI-CARE Agreement - Open Access funding for this article was provided by Recordati Rare Diseases Italy S.r.l. The funder had no role in the preparation, review, or approval of the manuscript.”

The original article has been corrected.

